# Ultra-Fast Degradation of *p*-Aminophenol by a Nanostructured Iron Catalyst

**DOI:** 10.3390/molecules23092166

**Published:** 2018-08-28

**Authors:** Rocio Benavente, David Lopez-Tejedor, Carlos Perez-Rizquez, Jose M. Palomo

**Affiliations:** Department of Biocatalysis, Institute of Catalysis (CSIC), Cantoblanco Campus UAM, Marie Curie 2, 28049 Madrid, Spain; r.benavente@csic.es (R.B.); david.lopez@csic.es (D.L.-T.); c.p.rizquez@csic.es (C.P.-R.)

**Keywords:** iron nanocatalyst, 4-aminophenol, environmental remediation, *Mentha* x *piperita*

## Abstract

Full degradation of *p*-aminophenol in aqueous solution at room temperature by using a heterogeneous nanostructured iron hybrid catalyst in the presence of hydrogen peroxide is described. A nanostructured iron catalyst was prepared by in situ formation of iron carbonate nanorods on the protein network using an aqueous solution of an enzyme, lipase B from *Candida antarctica* (CAL-B). A second kind of iron nanostructured catalyst was obtained by the sunsequent treatment of the hybrid with an aqueous liquid extract of *Mentha* x *piperita*. Remarkable differences were observed using TEM imaging. When *M. piperita* extract was used, nanoparticles appeared instead of nanorods. Catalytic activity of these iron nanocatalysts was studied in the degradation of the environmental pollutant *p*-aminophenol (pAP) under different operating parameters, such as pH, presence of buffer or hydrogen peroxide concentration. Optimal conditions were pH 4 in acetate buffer 10 mM containing 1% (*v*/*v*) H_2_O_2_ for FeCO_3_NRs@CALB, while for FeCO_3_NRs@CALB-*Mentha*, water containing 1% (*v*/*v*) H_2_O_2_, resulted the best. A complete degradation of 100 ppm of pAP was achieved in 2 and 3 min respectively using 1 g Fe/L. This novel nanocatalyst was recycled five times maintaining full catalytic performance.

## 1. Introduction

*para*-Aminophenol (pAP) is an important compound with an broad range of industrial application as a raw material in the petroleum, rubber, dye, medicine and photographic industries. It is also a well-known hazardous environmental pollutant [1,2]. In particular, pAP is a direct intermediate in the synthesis of paracetamol, so pAP contamination of the environment is possible due to paracetamol degradation [3]. Skin, eyes and respiratory system irritation, and also detrimental effects in blood and kidneys are some of the described symptoms to pAP exposure [4]. Therefore, a pAP concentration of 50.0 ppm has been established by the EU and the US as the maximum limit in paracetamol preparation [5]. Due to pAP toxicity, both to animals and the environment, pAP is a major environmental remediation issue.

Among the different approaches described for the degradation of this organic pollutant, the development and use of metal nanostructured materials has increased in recent years [6,7,8]. The high surface-to-volume ratio of nanomaterials compared to bulk materials, together with the advantages of being a heterogeneous phase, makes them attractive candidates for their application as catalysts [9,10,11,12]. In the other hand, iron is the most abundant metal in the planet, making it relatively inexpensive. In comparison with precious metals, iron is relatively nontoxic (i.e., it is considered by the regulatory authorities a “metal with minimum safety concern) [13]. Thus, iron (Fe) is extremely suitable for the elimination of environmental organic pollutants.

Several strategies have been described in the preparation of iron nanostructures. Depending on the metal source and experimental conditions, different iron species and nanostructures are obtained [14,15,16,17,18]. The most commonly obtained iron species nanoparticles are iron oxides like hematite (α-Fe_2_O_3_), maghemite (γ-Fe_2_O_3_), magnetite (Fe_3_O_4_), iron oxyhydroxide (FeOOH) and in particular cases, α-Fe in the form of nanoparticles, nanorods or even nanowires [19,20,21,22]. 

Here we present the preparation of a new type of iron nanostructured species: iron carbonate nanorods and nanoparticles. Well-dispersed iron nanostructures were synthesized in situ at room temperature in aqueous media by using an enzyme (*Candida antarctica* lipase B, CAL-B) as a biological matrix. The protein acted as stabilizing agent, also allowing the formation of a heterogeneous nanomaterial. A subsequent treatment of the formed nanohybrid with a liquid extract of *Mentha* x *piperita* was proposed as a variation of the original protocol. In the literature there are several examples of the use of plant extracts for the direct synthesis of nanoparticles [23,24]. However, to our knowledge, there are no precedents of protein-mediated coupled to the use of plant extracts in the synthesis of iron nanostructures. These methodologies represent a green alternative to conventional methods which involve the application of harsh conditions (e.g., high temperatures or the presence of organic solvents) and the necessity of highly controllable conditions or the utilization of special equipment [25,26]. Both iron nanohybrids were evaluated as catalysts in the degradation of *p*-aminophenol (100 mg/L) in aqueous media containing hydrogen peroxide. Different parameters of the degradation process were evaluated (pH, buffer presence, H_2_O_2_ concentration).

## 2. Results and Discussion

### 2.1. Synthesis of Nanostructured Iron Carbonate Biohybrids

**Figure 1:**
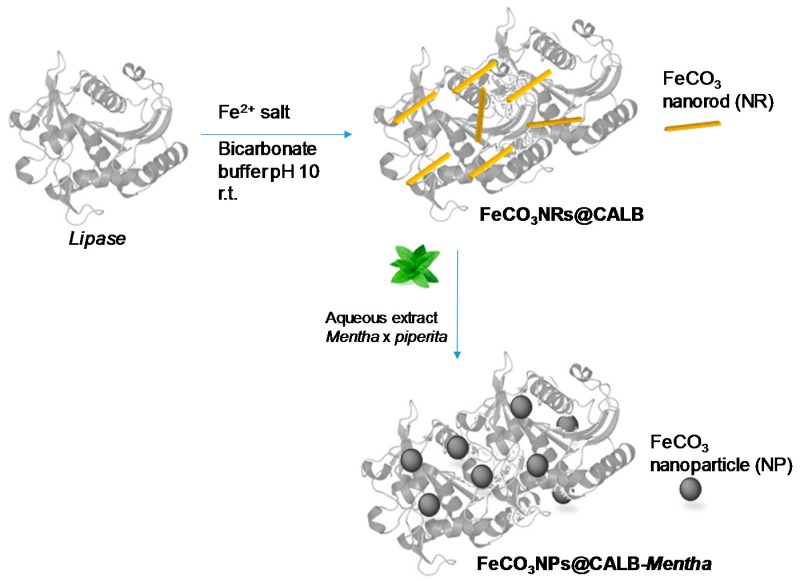
Biosynthesis of the iron carbonate nanorods, FeCO_3_NRs@CALB composites.

The synthesis of the bionanohybrid catalyst was performed by the combination of an aqueous solution of lipase B from *Candida antarctica* with a fully aqueous soluble iron salt (Mohr´s salt) at room temperature and under gentle stirring (Figure 1). 

In order to control the pH of the final solution -which must be higher than the isoelectric point of the lipase pI = 6 to obtain a negatively charged protein and ≤10 to avoid iron oxide nanoparticle formation in solution-, different buffers and different concentrations were tested, with 100 mM of sodium bicarbonate (pH 10) being identified as the best option. Then, commercial CAL-B solution (3.6 mL containing approx. 18 mg protein calculated by Bradford assay [27]) were dissolved in 60 mL of 100 mM of sodium bicarbonate (pH 10) and 600 mg of (NH_4_)_2_Fe(SO_4_)_2_ in solid form were added to the protein solution. At this iron salt concentration (10 mg/mL), the solution started to turn cloudy (first step in the bionanohybrid formation) after 30 min with a decrease in the pH of the solution to around 8, which was conserved unaltered during all the incubation time. After 16-h of incubation, a solid was obtained, washed several times with distilled water, centrifuged and lyophilized overnight. ICP-OES analysis revealed that this new bionanohybrid contained 47 wt. % of iron. 

**Figure 2:**
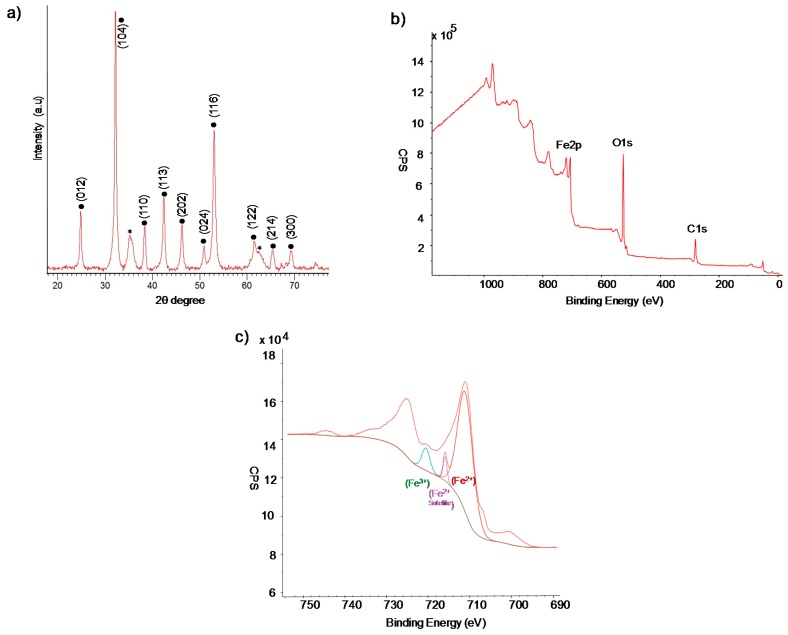
X-ray characterization of bionanohybrid. (**a**) XRD pattern (● FeCO_3_, * iron oxide impurity.). (**b**) XPS spectrum. (**c**) XPS Fe2p spectrum.

X-ray diffraction (XRD) analysis of the solid demonstrated the presence of iron (II) carbonate (siderite, FeCO_3_) as the main iron species, which is in concordance with previously described results [28,29], although some minor contamination with iron oxide (magnetite or maghemite) was found (Figure 2a). X-ray photoelectron spectroscopy (XPS) analysis of the solid confirmed the iron species, specially the presence of Fe(III) in the sample (Figure 2b,c). 

**Figure 3:**
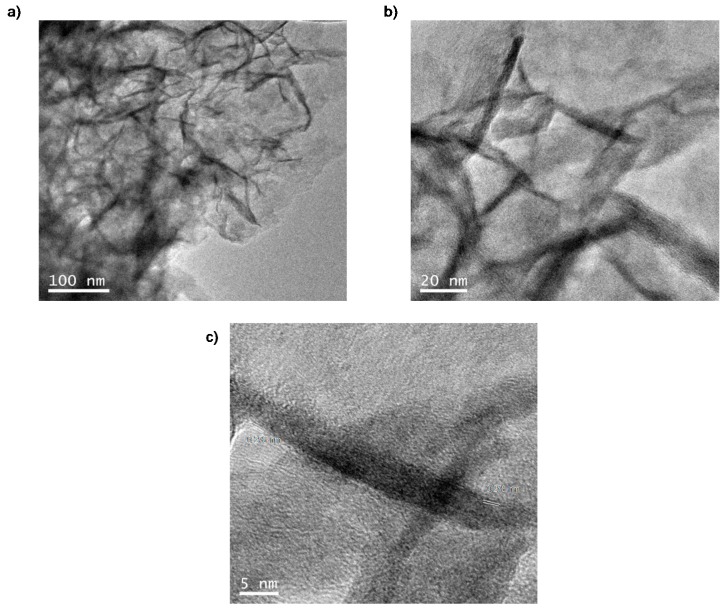
TEM analysis of FeCO_3_NRs@CALB. (**a**,**b**) TEM. (**c**) HRTEM.

TEM analysis of the solid revealed the formation of unexpected nanorods (NRs) of iron carbonate with a size of approx. 7 nm diameter × 59 nm long induced by the protein matrix, obtaining the so-called FeCO_3_NRs@CALB bionanohybrid (Figure 3).

Considering the advantages of the use of plant extract in the stabilization or reduction of different metals [23,24], a second strategy was developed using an aqueous extract of *Mentha* x *piperita.*

The synthesis of the bionanohybrid was performed as previously described in this section. Thus, 3.6 mL of commercial CAL-B solution were dissolved in 60 mL of 100 mM of sodium bicarbonate (pH 10), 600 mg of (NH_4_)_2_Fe (SO_4_)_2_ were added and the mixture was incubated for 16 h. After this time, the mixture was centrifuged and the supernatant removed. Then, the solid was added to 60 mL of an aqueous *Mentha* extract and the mixture was incubated for 30 min. The solution rapidly turned black as well as the solid. Then, the solid was centrifuged, washed and lyophilized for 16 h, affording a black solid. 

**Figure 4:**
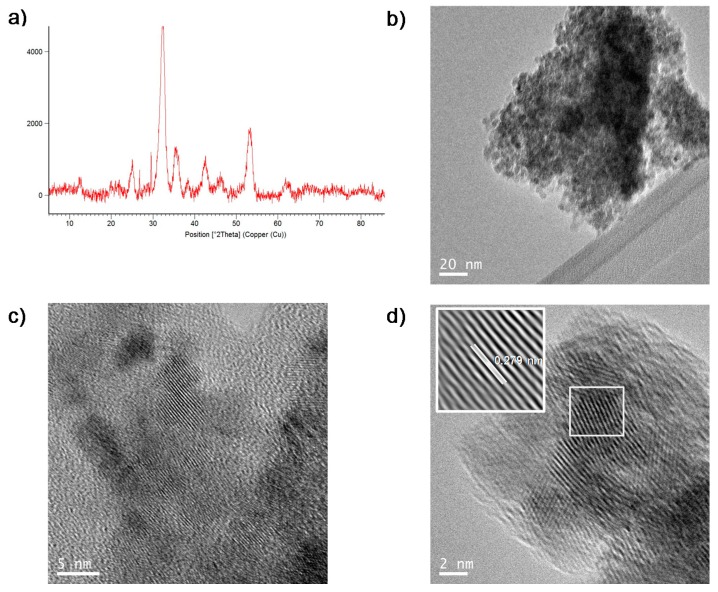
Characterization of FeCO_3_NRs@CALB-*Mentha.* (**a**) XRD. (**b**) TEM. (**c**) HRTEM. (**d**) HRTEM (inset IFFT).

This new solid, contained the same amount of Fe (47%, measured by ICP) as the FeCO_3_NRs@CALB. XRD analysis showed that this treatment with the plant extract—the only difference with the synthetic protocol of FeCO_3_NRs@CALB—modified the iron species of the sample (Figure 4a). Although the main iron species in the solid were the same than in FeCO_3_NRs@CALB, siderite, the amount of iron oxide increased (Figure 4a). 

TEM analyses revealed the formation of nanoparticles of diameter size around 4–5 nm instead of nanorods (Figure 4b–d). HRTEM showed regular lattice fringes with an interplanar spacing of 0.279 nm in nanoparticles (Figure 4d), which correspond to the (104) lattice planes of siderite [30]. The interplanar spacing in nanorods from FeCO_3_NRs@CALB was the same (Figure 3). 

The treatment with *Mentha* extract, which in principle was used to act as reducing agent [23], seems to change the iron species reducing the size of the iron nanostructures formed from nanorods to nanoparticles. It has been described that for iron nanostructures the particular methodology is a critical step in order to obtain different morphologies [14,15]. 

**Figure 5:**
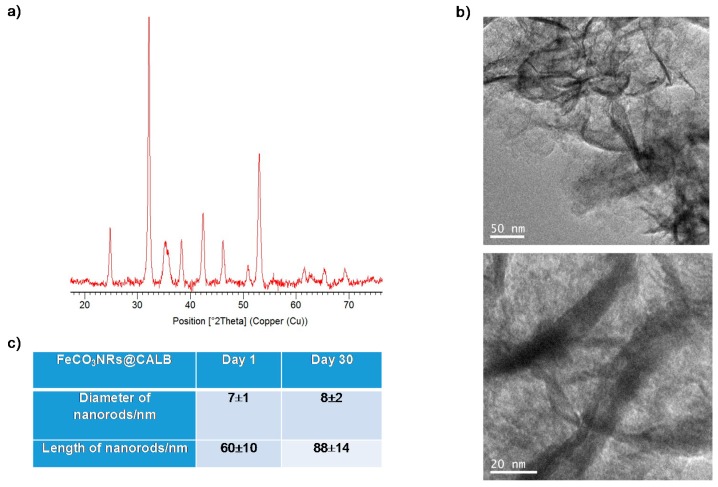
Characterization of FeCO_3_NRs@CALB after 30 days. (**a**) XRD pattern nanocomposite. (**b**) TEM images of the nanocomposite. (**c**) Comparison of nanorods size of the nanocomposite at day 1 and day 30 after synthesis.

The FeCO_3_NRs@CALB bionanohybrid was stored at room conditions for 30 days to evaluate its stability. After that, XRD image showed that no significant changes were observed in the iron species (Figure 5). TEM images revealed a slight increase in the width and the length of the nanorods, increasing the latter from around 60 nm to 88 nm (Figure 5). 

### 2.2. Degradation of pAP Catalyzed by FeCO_3_NRs@CALB

**Figure 6:**
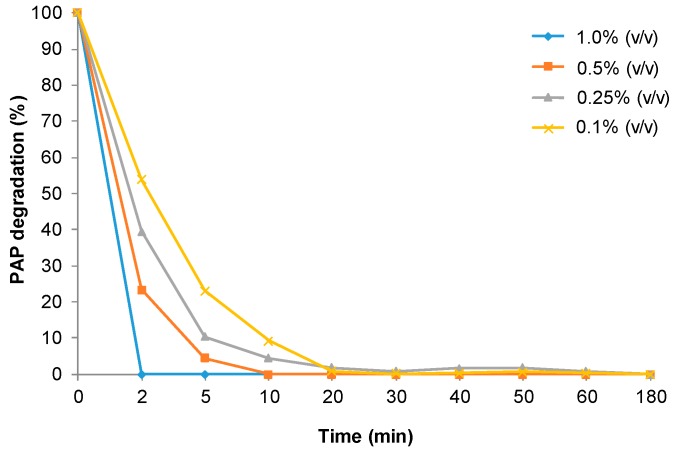
Profile of pAP degradation in acetate buffer at pH 4 containing different amount of H_2_O_2_ catalyzed by FeCO_3_NRs@CALB.

FeCO_3_NRs@CALB biohybrid was used as catalyst in the degradation of pAP (100 mg/L). First, substrate was solubilized in distilled water (pH around 7) and nanocatalyst (3 mg) were added to 2 mL of pAP solution. Under these conditions, containing 1% (*v*/*v*) of H_2_O_2_ at room temperature, more than 95% degradation of pAP was observed after 10 min. Similar results were obtaining using 5 mM phosphate buffer at 7 as solvent. To evaluate the effect of pH in the catalytic performance, the reaction was repeated using pAP previously dissolved in sodium acetate buffer pH 4 (Figure 6). Iron nanocatalysts were faster at these conditions, and pAP was degraded in 2 min in the presence of 1% (*v*/*v*) of H_2_O_2_. Importantly, no traces of any compounds were detected by HPLC after 50 min.

Considering the rate obtained at this pH, the amount of H_2_O_2_ was evaluated. The reaction was slower in the presence of 0.5% (*v*/*v*) H_2_O_2_ and full degradation was achieved after 10 min. Using less amount of oxidant, the catalytic performance of FeCO_3_NRs@CALB was reduced and pAP degradation was not complete before 30 min (Figure 6).

### 2.3. Degradation of pAP Catalyzed by FeCO_3_NPs@CALB-Mentha

**Figure 7:**
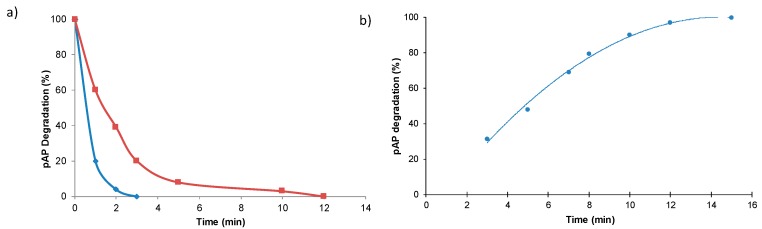
Profile of pAP degradation in water containing 1% (*v*/*v*) of H_2_O_2_ catalyzed by bionanohybrids. (**a**) Comparison between FeCO_3_NRs@CALB (red line) and FeCO_3_NPs@CALB-*Mentha* (blue line) at 2 mL reaction volume. (**b**) FeCO_3_NPs@CALB-*Mentha* at 10 mL reaction volume. The amount of catalyst was 3 mg in all cases.

The catalytic capacity of the new biohybrid FeCO_3_NPs@CALB-*Mentha* was also tested in the catalytic degradation of pAP (100 mg/L) using 1% (*v*/*v*) H_2_O_2_. The reaction was very fast in distilled water, degrading all pAP in 3 min, being almost 4-fold faster than the FeCO_3_NRs@CALB biohybrid under these conditions (Figure 7a). The reaction was also performed using 0.2 g of catalyst per L of the reaction volume_._ At these conditions, pAP was completely degraded in 15 min (Figure 7b).

Then, catalytic capacity of this biohybrid was tested under different pHs (Table 1). In this case, acidic conditions (pH 4) were tested using acetate buffer (10 or 100 mM) or directly in acidic water pH adjusted using HCl. Tap water was also used for the reaction, giving similar results to those obtained using distilled water (Table 1).

FeCO_3_NPs@CALB-*Mentha* showed less catalytic activity at lower pH, and around 80% degradation was obtained after 20 min at pH 4 acetate buffer (Table 1, entries 1–2). The use of acidic water even resulted in worse conversion values (Table 1, entry 3). 

Considering the low effect of buffer presence on the biohybrid, 0.5 mM phosphate buffer was used to adjust pH to 6 or 7. Reaction was slightly faster at pH 7 than pH 6 with complete substrate degradation in 15 min, similar results that those obtained using distilled or tab water (Table 1).

**Figure 8:**
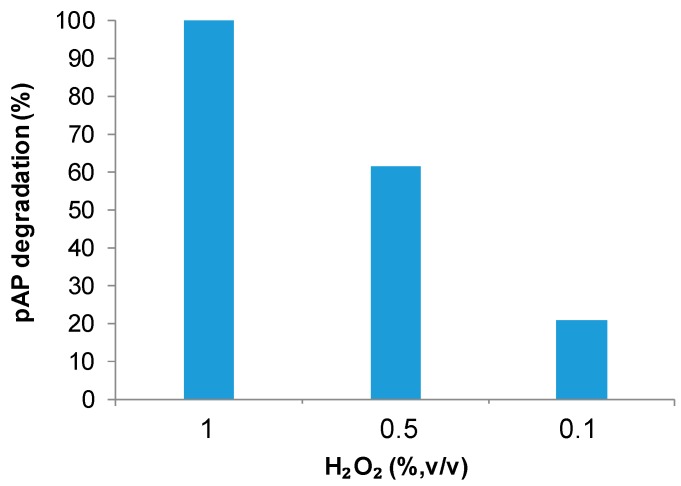
Effect of the amount of hydrogen peroxide in the pAP degradation catalyzed by hydrogen peroxide. Reaction conditions were 3 mg catalyst, 10 mL of pAP solution in distilled water (100 mg/L), room temperature for 15 min.

Therefore, optimal condition for the degradation of 100 ppm of pAP catalyzed by FeCO_3_NPs@CALB-*Mentha* was obtained using water as solvent, whereas for FeCO_3_NRs@CALB was using acetate (Figure 6). In both cases, the mechanism of degradation was similar to the previous reported, where pAP was oxidized to hydroquinone (HQ) and p-benzoquinone and finally the benzene rings are opened and oxidized to other smaller compounds which finally most of them are degraded to CO_2_ and H_2_O [6,31]. Considering the fast reaction, the effect of hydrogen peroxide concentration was again also tested with this catalyst (Figure 8). 

However, like the previous results with FeCO_3_NRs@CALB, the catalytic capacity of the nanobiohybrid decreased 40% with the addition of 0.5% (*v*/*v*) H_2_O_2_ and even more than 80% with adding 0.1% (*v*/*v*).

**Figure 9:**
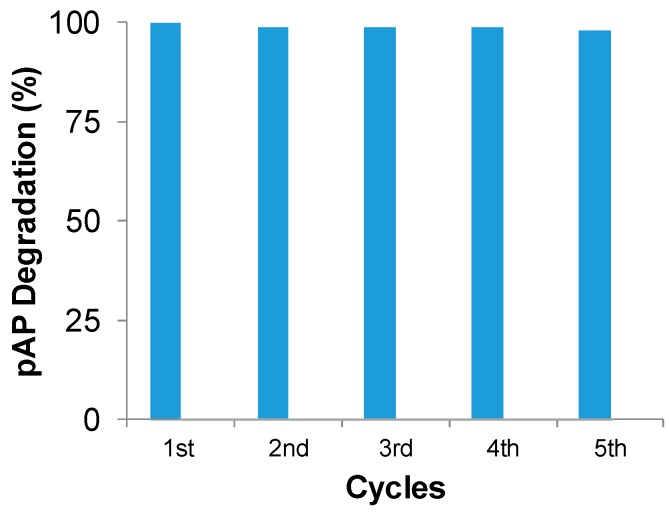
Reuse of FeCO_3_NPs@CALB-*Mentha* in the degradation of pAP. Reaction conditions were: 3 mg catalyst, 2 mL of pAP solution in distilled water (100 mg/L), room temperature for 3 min.

Finally, to evaluate the possible industrial applicability of this catalyst, a recycling process was tested under optimal conditions: distilled water as solvent and 1% (*v*/*v*) hydrogen peroxide (Figure 9). Catalyst was used for five cycles in the degradation process without loss of catalytic performance.

Thus, these new nanocatalysts constitute a very promising alternative for the degradation of organic pollutants, especially pAP, where in many cases these results improve the efficiency in terms of time of degradation and amount of sample achieved using other catalysts described in the literature for this reaction [7,8]. 

## 3. Materials and Methods 

*Candida antarctica* lipase B (CAL-B) solution was from Novozymes (Copenhagen, Denmark). Ammonium iron(II) sulfate hexahydrate [(NH_4_)_2_Fe(SO_4_)_2_ × 6H_2_O (Mohr´s salt)], hydrogen peroxide (33%), *p*-aminophenol, sodium bicarbonate and sodium borohydride were purchased from Sigma-Aldrich (St. Louis, MO, USA). HPLC grade acetonitrile was purchased from Scharlab (Barcelona, Spain). Tap water came from the Canal de Isabel II (Madrid Region, Spain).

Inductively coupled plasma atomic emission spectrometry (ICP-AES) was performed on a OPTIMA 2100 DV instrument (PerkinElmer, Waltham, MA, USA). X-Ray diffraction (XRD) patterns were obtained using a Texture Analysis D8 Advance Diffractometer (Bruker, Billerica, MA, USA) with Cu Kα radiation. X-ray photoelectron analysis (XPS) was carried out on SPECS GmbH (Berlin, Germany) spectrometer equipped with a Phoibos 150 9MCD energy analyzer. A non-monochromatic aluminum X-ray source with a power of 200 W and voltage of 12 kV was used using as reference standard the C1s adventitial carbon 284.8 eV. Transmission electron microscopy (TEM) and high resolution TEM microscopy (HRTEM) images were obtained on a 2100F microscope (JEOL, Tokyo, Japan) equipped with an EDX detector INCA x-sight (Oxford Instruments, Abingdon, UK). Interplanar spacing in the nanostructures was calculated by using the inversed Fourier transform with the GATAN digital micrograph program (Corporate Headquarters, Pleasanton, CA, USA). Scanning electron microscopy (SEM) imaging was performed on a TM-1000 microscope (Hitachi, Tokyo, Japan). To recover the biohybrids, a Biocen 22 R (Orto-Alresa, Ajalvir, Spain) refrigerated centrifuge was used. Spectrophotometric analyses were run on a V-730 spectrophotometer (JASCO, Tokyo, Japan). A spectrum P100 HPLC system (Thermo Scientifics, Waltham, MA, USA) was used. Analyses were run at 25 °C using an L-7300 column oven (Hitachi, Tokyo, Japan) and a UV6000LP detector (Thermo Scientifics, Waltham, MA, USA).

### 3.1. Synthesis of Nanostructured FeCO_3_NRs@CALB Hybrid

Commercial *Candida antarctica* lipase B solution (3.6 mL, containing 4 mg lipase/mL) was added to 60 mL sodium bicarbonate buffer 0.1 M pH 10 in a 100 mL glass bottle containing a small magnetic bar stirrer (12 × 4.5 mm). The solution was stirred in a magnetic agitator at 380 rpm (*this is important to avoid iron oxidation)* for 2 min. Then Fe(NH_4_)_2_(SO_4_)_2_·6H_2_O (600 mg, 10 mg/mL) was added to the protein solution, while maintaining the stirring. This was continued for 16 h at room temperature. After the first 30 min of incubation, the solution turned cloudy (greenish-gray) and the pH decreased from 10 to 7–8. After 16 h of incubation, the solution turned very dark green. Then, the mixture was centrifuged at 8000 rpm for 5 min, adding 11 mL per each 15 mL Falcon-type tube. The generated pellet was resuspended in 15 mL of distilled water and centrifuged again at 8000 rpm for 5 min and the supernatant removed. This process was repeated once more. Finally, the supernatant was removed, and the pellet of each Falcon tube was resuspended in 2 mL of water, all solutions combined in a round-bottomed flask, frozen with liquid nitrogen and lyophilized for 16 h. Characterization of the bionanohybrid was performed by XRD, XPS, SEM and TEM analysis. The bionanohybrid was again characterized after 1 month of preparation. 

### 3.2. Preparation of the Extracted Aqueous Solution of Mentha x Piperita

Dry leaves of *Mentha* x *piperita* (10 g, purchased from SoriaNatural, Garray, Spain) were added to 100 mL of previously heated bi-distilled water (at 100 °C). This mixture was boiled for 10 min. Then, the brown dark solution obtained was recovered by centrifugation (10,000 rpm) and filtration. To separate plant material from the aqueous solution, the mixture was transferred to centrifuge tubes and centrifuged at 10,000 rpm for 10 min at 12 °C. Supernatant was collected and filtered using filter paper (Prat Dumas, Couze-et Saint Front, France) to completely remove any remaining solid. Liquid plant extract was used immediately after preparation.

### 3.3. Synthesis of Nanostructured FeCO_3_NPs@CALB-Mentha Biohybrid.

Commercial *Candida antarctica* lipase B solution (3 mL) was added to sodium bicarbonate buffer (60 mL, 0.1 M, pH 10) in a 100 mL glass bottle containing a small magnetic bar stirrer (12 × 4.5 mm). The solution was stirred on a magnetic agitator at 380 rpm for 2 min. Then Fe(NH_4_)_2_(SO_4_)_2_· 6H_2_O (600 mg, 10 mg/mL) were added to the protein solution, while maintaining the stirring. After 16 h of incubation at room temperature, the solution turned very dark green. Then, the mixture was centrifuged at 8000 rpm for 5 min, and the supernatant was discarded. The solid was dissolved in 60 mL of an aqueous extract of *Mentha* x *piperita* for 30 min. The solution turned black immediately after the addition of the *Mentha* extract. Then the mixture was centrifuged at 8000 rpm for 5 min. The supernatant was discarded and pellet was resuspended in 15 mL of water. It was centrifuged again at 8000 rpm for 5 min and the supernatant removed. The process was repeated once more. Finally, the recovered pellet of each Falcon was resuspended in 2 mL of water. Solutions were frozen with liquid nitrogen and lyophilized for 16 h. Characterization of the novel iron nanostructured hybrid was performed by XRD and TEM analysis. 

### 3.4. Catalytic Degradation of p-Aminophenol by Iron Nanostructured Catalyst 

pAP (2 mg) was dissolved in solutions (18.88 mL) of distilled water, acidic water pH 4, acetate buffer (pH 4) or phosphate buffer (pH 6.7) and different amount of hydrogen peroxide (%, *v*/*v*) (from 0.02 to 0.22 mL) were added. To initialize the reaction, 2 or 10 mL of this solution were added to a glass bottle containing 3 mg of bionanohybrid and stirred gently at room temperature on an orbital shaker (320 rpm). In the case of using FeCO_3_NPs@CALB-*Mentha*, the reaction was performed using 3 mg of catalyst in 10 mL of pAP 1 mM solution. Experiments were performed in triplicate.

At different times samples (80 µL) were taken and the reaction was followed by HPLC. Samples were first centrifuged at 8000 rpm for 5 min and then 50 µL were diluted 40 times in bi-distilled water before injection. HPLC column was C8 Kromasil 150 × 4.6 mm AV-2059. HPLC conditions were: an isocratic mixture of 30% acetonitrile and 70% bi-distilled water, UV detection at 270 nm using a Diode array detector, and a flow rate of 0.6 mL/min. Under these conditions, retention times of pAP and H_2_O_2_ were 4.03 min, and 2.6 min respectively. The possible adsorption of substrate to the catalyst was first tested and without the presence of hydrogen peroxide no reaction was observed and the full area of the substrate was unaltered in the HPLC analysis.

### 3.5. Reuse of FeCO_3_NPs@CALB-Mentha Hybrid 

FeCO_3_NPs@CALB-*Mentha* catalyst was reused five cycles in the degradation of 100 ppm of pAP at optimal conditions: pAP in distilled water, 1% (*v*/*v*) hydrogen peroxide using 3 mg of catalyst in 2 mL of solution. A syringe with a filter was used to perform the reaction, removing the solution when finished while preventing leakage of the catalyst. An adjustment (around 10%) due to the negligible loss of catalyst through the filter was applied. No leaching of iron content of the catalyst was determined, even after the fifth cycle. 

## 4. Conclusions

Herein, we have described a very simple and efficient strategy to synthesize iron nanostructured catalysts. They were successfully applied in the ultra-fast full degradation of pAP in aqueous media. The biohybrid FeCO_3_NRs@CALB, containing iron nanorods, worked better at pH 4 whereas the FeCO_3_NPs@CALB-*Mentha*, containing iron nanoparticles, was better at pH 7. In both cases, 100 ppm of pAP was degraded around 2–3 min at 1 g cat/L or 15 min at 0.2 g cat/L. The nanobiohybrids were quite stable and could be recycled at least 5 times without any decrease in their catalytic capacity.

## Figures and Tables

**Table 1:** pAP degradation at different conditions catalyzed by FeCO_3_NRs@CALB-*Mentha* ^a^.

Solvent	(mM)	pH	Time (min)	pAP Degradation (%)
Acetate	100	4	20	81
Acetate	10	4	20	76
Adjusted Tap water	-	4	17	66
Phosphate	0.5	6	16	99
Phosphate	0.5	7	15	99
Tap water	-	7.4	16	99
Distilled H_2_O	-	7	15	99

^a^ Reaction conditions were: 3 mg catalyst, 10 mL of pAP solution in distilled water (100 mg/L), 1% (*v*/*v*) hydrogen peroxide, room temperature.
